# On Nervous Diseases

**Published:** 1851-04-01

**Authors:** 


### ON NERVOUS DISEASES.

Dr. Bennett (of Edinburgh), who has paid so much attention to the affections o the nervous system, and whose valuable microscopical researches into the nature of morbid nervous tissue are well known to the profession, has recently been delivering a course of clinical lectures (on nervous diseases) in the ward of the Royal Infirmary, Edinburgh. A condensed report of these lectures has been published in the March Number of the "*Monthly Journal of Medical Science*;" and from that able periodical we borrow the subjoined interesting and able observations.

"The diagnosis of these disorders is dependent on a kind of knowledge altogether different from that appertaining to the consideration of cutaneous, pulmonary, or cardiac affections. In these last, as we have seen, a direct appeal to the senses enables us to arrive at conclusions with tolerable accuracy. An arbitrary classification of skin diseases once established, with clear definitions, we have only to apply these to the appearances observed to ascertain the disorder. Once master the practical difficulty of distinguishing with exactitude moist from dry râles, whether a murmur replace the first or second sound of the heart, and what is its position, and we possess a key which, with the aid of percussion, will enable us to arrive at the certain diagnosis of pulmonary and cardiac affections. But, with regard to nervous diseases, no such exactitude is attainable in the present state of the science or art of medicine. The encephalon is an aggregation of various parts, more or less connected together, the functions of which are by no means determined. In health these act in harmony, but in disease they are so irregularly disordered that, while the action of one is excited, that of another may be perverted or annihilated. Thus, nothing is more common than to observe some of the most fatal nervous diseases, such as hydrophobia, leaving after death no lesion detectable by the most careful histological examination, whilst on other occasions tumours and extensive destruction of the cerebral mass may exist, without producing any effects whatever. And yet, notwithstanding the obvious difficulties which oppose themselves to exactitude of diagnosis, careful observation, conjoined with a knowledge of physiology and pathology, will enable us to approximate closely towards, if not actually reach, a correct opinion in the great majority of cases. Seeing, then, the necessity of possessing a knowledge of the general principles arrived at by physiologists and pathologists, in order that we may form a correct diagnosis at the bedside, I propose, in the first instance, placing these shortly before you.

"I. *Structure and Arrangement of the Nervous System.*—To the eye, the nervous system appears to be composed of two structures,—the grey or ganglionic, and the white or fibrous. The ganglionic, when examined under high powers, may be seen to be composed of nucleated corpuscles, varying greatly in size and shape, mingled with a greater or less number of nerve tubes, also varying in calibre. The important fact, with regard to these, is, that many of the corpuscles may be demonstrated to throw out prolongations, which are in direct communication with, or constitute, the central band or axis of Remak and Purkinje within the tubes. The fibrous structure may be shown to consist of minute tubes, which are smallest towards the periphery of the cerebrum, larger towards its base, and largest in the nerves. They are of three kinds, —1st, Cylindrical, as observed in the optic and auditory nerves; 2nd, Varicose, as seen in the white substance of the cerebral lobes and of the spinal cord; and 3rd, Of regular size throughout, as seen in the nerves. There are also bundles of gelatinous or flat fibres, the nature of which is much disputed, very common in the olfactory nerve and sympathetic system of nerves. There can be no doubt that some nerve tubes run into the ganglionic corpuscles, whilst others originate from them (Wagner, Kölliker.) It is even possible that the same ganglionic corpuscle may receive and give off nerve tubes, each having distinct properties, the one of conveying the influence of impressions to, and the other of conveying influences from, the nervous centres. The peripheral termination of the nerves is in loops or arcs.

"The general arrangement of the two kinds of structures should be known. By cerebrum, or brain proper, ought to be understood that part of the encephalon constituting the cerebral lobes, situated above and outside the corpus callosum; by the spinal cord, all the parts situated below this great commissure, consisting of corpora striata, optic thalami, corpora quadrigemina, cerebellum, pons Varolii, medulla oblongata, and medulla spinalis. In this way, we have a cranial and a vertebral portion of the spinal cord.

"In the cerebrum, or brain proper, the ganglionic or corpuscular structure is external to the fibrous or tubular. It presents on the surface numerous anfractuosities, whereby a large quantity of matter is capable of being contained in a small space. This crumpled up sheet of grey substance has been appropriately called the hemispherical ganglion. (Solly.) In the cranial portion of the spinal cord, the grey matter exists in masses, constituting a chain of ganglia at the base of the encephalon, more or less connected with each other and with the white matter of the brain proper above, and the vertebral portion of the cord below. In this last part of the nervous system the grey matter is internal to the white, and assumes the form of the letter X, having two posterior and two anterior cornua,—an arrangement which allows the latter to be distributed in the form of nerve tubes to all parts of the frame.

"The white tubular structure of the vertebral portion of the cord is divided by the anterior and posterior horns of grey matter, together with the anterior and posterior sulci, into three divisions or columns on each side. On tracing these upwards into the medulla oblongata, the anterior and middle ones may be seen to decussate with each other, whilst the posterior columns do not decussate. On tracing these up into the cerebral lobes, we observe that the anterior columns, or pyramidal tracts, send off a bundle of fibres, which passes below the olivary body, and is lost in the cerebellum—(*Arciform band* of Solly). The principal portion of the tract passes through the corpus striatum, and anterior portion of the optic thalamus and is ultimately, lost in the white substance of the cerebral hemispheres. The middle column, or olivary tract, may be traced through the substance of the optic thalamus and corpora quadrigemina, to be in like manner lost in the cerebral hemispheres. The posterior column, or restiform tract, passes almost entirely to the cerebellum. In addition to the diverging fibres in the cerebral hemispheres which may be thus traced from below upwards, connecting the hemispherical ganglion with the structures below, the brain proper also possesses bands of transverse fibres, constituting the commissures connecting the two hemispheres of the brain together, as well as longitudinal fibres connecting the anterior with the posterior lobes.

"II. *Functions of the Nervous System.*—The great difference in structure existing between the grey and white matter of the nervous system, would *à priori* lead to the supposition that they performed separate functions. The theory at present entertained on this point is, that, while the grey matter eliminates or evolves nervous power, the white matter simply conducts to and from this ganglionic structure the influences which are sent or originate there.

"The brain proper furnishes the conditions necessary for the manifestation of the intellectual faculties properly so called, of the emotions and passions, of volition, and is essential to sensation. That the evolution of the power especially connected with mind is dependent on the hemispherical ganglion is rendered probable by the following facts:—1. In the animal kingdom generally, a correspondence is observed between the quantity of grey matter, depth of convolutions, and the sagacity of the animal. 2. At birth, the grey matter of the cerebrum is very defective, so much so, indeed, that the convolutions are, as it were, in the first stage of their formation, being only marked out by superficial fissures almost confined to the surface of the brain. As the cineritious substance increases, the intelligence becomes developed. 3. The results of experiments by Flourens, Rolando, Hertwig, and others, have shown that, on slicing away the brain, the animal becomes more dull and stupid in proportion to the quantity of cortical substance removed. 4. Clinical observation points out, that in those cases in which the disease has been afterwards found to commence at the circumference of the brain and proceed towards the centre, that the mental faculties are affected *first;* whereas in those diseases which commence at the central parts of the organ and proceed towards the circumference, they are affected *last.*

"The white tubular matter of the brain proper serves, by means of the diverging fibres, to conduct the influences originating in the hemispherical ganglion to the nerves of the head and trunk, whilst they also conduct the influence of impressions made on the trunk, in an inverse manner, up to the cerebral convolutions. The other transverse and longitudinal fibres which connect together the two hemispheres, and various parts of the hemispherical ganglion, are probably subservient to that combination of the mental faculties which characterizes thought.

"The spinal cord, both in its cranial and vertebral portions, furnishes the conditions necessary for combined movements; and that the nervous power necessary for this purpose depends upon the grey matter, is rendered probable by the following facts:—1st, Its universal connexion with all motor nerves. 2nd, Its increased quantity in those portions of the spinal cord from whence issue large nervous trunks. 3rd, Its collection in masses at the origin of such nerves in the lower animals as furnish peculiar organs requiring a large quantity of nervous power, as in the triglia volitans, raia torpedo, silurus, &c. 4th, Clinical observation points out that, in cases where the central portion of the cord is affected previous to the external portion, an individual retains the sensibility of, and power of moving, the limbs, but wants the power to stand, walk, or keep himself erect, when the eyes are shut; whereas, when diseases commence in the meninges of the cord or externally, pain, twitchings, spasms, numbness, or paralysis, are the symptoms present, dependent on lesion of the white conducting matter.

"The white matter of the cord acts as a conductor, in the same manner that it does in the brain proper, and there can be no doubt that the influence arising from impressions is carried along the tracts, formerly noticed, which connect the brain and two portions of the spinal cord together. It is now also determined, that many of the fibres in the nerves may be traced directly into the grey substance of the cord—a fact originally stated by Grainger, but confirmed by Budge and Kölliker.

"The various nerves of the body consist for the most part of nerve tubes, running in parallel lines. Yet some contain ganglionic corpuscles, as the olfactory, and the expansion of the optic nerve constituting the retina, whilst the sympathetic nerve contains in various places, not only ganglia, but gelatinous flat fibres. The posterior roots of the spinal nerves possess a ganglion, the function of which is quite unknown. These roots are connected with the posterior horn of grey matter in the cord, while the anterior roots are connected with the anterior horns. As regards function, the nerves may be considered as—1st, Nerves of special sensation, such as the olfactory, optic, auditory, part of the glosso-pharyngeal and lingual branch of the fifth. 2nd, Nerves of common sensation, such as the greater portion of the fifth, and part of the glosso-pharyngeal. 3rd, Nerves of motion, such as the third, fourth, lesser division of the fifth, sixth, facial or portio dura of the seventh, and the hypo-glossal. 4th, Senso-motory or mixed nerves, such as the pneumo-gastric, the accessory, and the spinal nerves. 5th, Sympathetic nerves, including the numerous ganglionic nerves of the head, thorax, and abdomen,—the exact function of which has not been determined.

"All nerves are endowed with a peculiar vital property, called sensibility, inherent in their structure, by virtue of which they may be excited on the application of appropriate stimuli, so as to transmit the influence of the impressions they receive to or from the brain, spinal cord, or certain ganglia, which may be considered as nervous centres. The nerves of special sensation convey *to* their nervous centres the influence of impressions caused by odoriferous bodies, by light, sound, and by sapid substances. The nerves of common sensation convey the influence of impressions *to* their nervous centres, caused by mechanical or chemical substances. The nerves of motion carry *from* the nervous centres the influence of impressions whether psychical or physical. (Todd.) The mixed nerves carry the influence of stimuli both to and from, combining in themselves the functions of common sensation and of motion. Although the sympathetic nerves also undoubtedly carry the influences of impressions, the direction of these cannot be ascertained, from their numerous anastomosis, as well as from the ganglia scattered over them, all of which act as minute nervous centres. But there *are* cases where certain psychical stimuli (as the emotions) act on organs through these nerves, and where certain diseases (as colic, gall-stones, &c.) excite through them sensations of pain.

"Sensation may be defined to be *the consciousness of an impression*, and that it may take place, it is necessary,—1st, That a stimulus should be applied to a sensitive nerve, which produces an impression; 2nd, That, as the result of this impression, a something should be generated, which we call an influence, which influence is conducted along the nerve to the hemispherical ganglion; 3rd, On arriving there, it calls into action that faculty of the mind called consciousness or perception, and sensation is the result. It follows that sensation may be lost by any circumstance which destroys the sensibility of the nerve to impressions, which impedes the process of conducting the influence generated by these impressions; or, lastly, which renders the mind unconscious of them. Illustrations of how sensation may be affected in all these ways must be familiar to you, from circumstances influencing the ultimate extremity of a nerve, as on exposing the foot to cold,—from injury to the spinal cord, by which the communication with the brain is cut off, or from the mind being inattentive, excited, or suspended.

"The independent endowment of nerves is remarkably well illustrated by the fact, that whatever be the stimulus which calls their sensibility into action, the same result is occasioned. Mechanical, chemical, galvanic, or other *physical* stimuli, when applied to the course or the extremities of a nerve, cause the very same results as may originate from suggestive ideas, perverted imagination, or other *psychical* stimuli. Thus a chemical irritant, galvanism, or pricking and pinching a nerve of motion, will cause convulsion and spasms of the muscles to which it is distributed. The same stimuli applied to a nerve of common sensation will cause pain, to the optic nerve flashes of light, to the auditory nerve ringing sounds, and to the tip of the tongue peculiar tastes. Again, we have lately had abundant opportunities of seeing that suggestive ideas, or stimuli arising in the mind, may induce peculiar effects on the muscles, give rise to pain or insensibility, and cause perversion of all the special senses.

"Motion is accomplished through the agency of muscles, which are endowed with a peculiar vital property, called contractility, in the same way that nerve is endowed with the property of sensibility. Contractility may be called into action altogether independent of the nerves (Haller), as by stimulating an isolated muscular fasciculus directly. (Weber.) It may also be excited by physical or psychical stimuli, operating through the nerves. Physical stimuli applied to the extremities or course of a nerve, may cause convulsions of the parts to which the motor filaments are distributed directly, or they may induce combined movements in other parts of the body *diastaltically* (Marshall Hall),—that is, through the spinal cord. In this latter case the following series of actions take place:—1st, The influence of the impression is conducted to the spinal cord by the afferent or *esodic* filaments which enter the grey matter; 2nd, A motor influence is transmitted outwards by one or more efferent or *exodic* nerves; 3rd, This stimulates the contractility of the muscles to which the latter are distributed, and motion is the result. Lastly, contractility may be called into action by psychical stimuli or mental acts—such as by the will and by certain emotions. Integrity of the muscular structure is necessary for contractile movements; of the spinal cord, for diastaltic or reflex movements; and of the brain proper, for voluntary or emotional movements.

"Thus, then, we may consider that the brain acting alone furnishes the conditions necessary for intelligence; the spinal cord acting alone furnishes the conditions essential for the co-ordinate movements necessary to the vital functions; and the brain and spinal cord acting together furnish the conditions necessary for voluntary motion and sensation.

"An account of the various cerebral, spinal, and cerebro-spinal functions, as they are performed separately or conjointly, belongs to the course of the Institutes of Medicine, and with these you are supposed to be familiar. It is important, however, that we dwell more at length on

"III. *The Pathological Laws which regulate Diseased Functions of the Nervous System.*—For the purposes of diagnosis and treatment, it is a matter of great importance to attend to the following generalizations:—

"(1.) *The amount of fluids within the cranium must always be the same so long as its osseous walls are capable of resisting the pressure of the atmosphere.* There are few principles in medicine of greater practical importance than the one we are about to consider,—the more so, as many able practitioners have lately abandoned their former opinions on this head, and on what I consider to be very insufficient grounds. On this point, therefore, I cannot do better than condense and endeavour to put clearly before you the forcible arguments of the late Dr. John Reid, with such other considerations as have occurred to myself.

"That the circulation within the cranium is different from that in other parts of the body, was first pointed out by the second Monro. It was tested experimentally by Dr. Kellie of Leith, ably illustrated by Dr. Abercrombie, and successfully defended by Dr. John Reid. The views adopted by these distinguished men were, that the cranium forms a spherical bony case, capable of resisting the atmospheric pressure, the only openings into it being the different foramina by which the vessels, nerves, and spinal cord pass. The encephalon, its membranes and bloodvessels, with perhaps a small portion of the cerebro-spinal fluid, completely fill up the anterior of the cranium, so that no substance can be dislodged from it without some equivalent in bulk taking its place. Dr. Munro used to point out, that a jar, or any other vessel similar to the cranium, with unyielding walls, if filled with any substance, cannot be emptied without air or some other substance taking its place. To use the illustration of Dr. Watson, the contents of the cranium are like beer in a barrel, which will not flow out of one opening, unless provision be made at the same time that air rushes in. The same kind of reasoning applies to the spinal canal, which, with the interior of the cranium, may be said to constitute one large cavity, incompressible by the atmospheric air.

"Before proceeding further, we must draw a distinction between pressure on, and compression of an organ. Many bodies are capable of undergoing a great amount of pressure without undergoing any sensible decrease in bulk. By compression must be understood, that a substance occupies less space from the application of external force, as when we squeeze a sponge, or compress a bladder filled with air. Fluids generally are not absolutely incompressible, yet it requires the weight of one atmosphere, or fifteen pounds in the square inch, to produce a diminution equal to $\frac{1}{20000}$th part of the whole. Now this is so exceedingly small a charge upon a mass equal in bulk to the brain, as not to be appreciable to our senses. Besides, the pressure on the internal surface of the bloodvessels never exceeds ten or twelve pounds on the square inch, during the most violent exertion, so that, under no possible circumstances, can the contents of the cranium be diminished even the $\frac{1}{20000}$th part. When the brain is taken out of the cranium, it may, like a sponge, be compressed, by squeezing fluid out of the bloodvessels; but during life, surrounded, as it is, by unyielding walls, this is impossible. For let us, with Abercrombie, say, that the whole quantity of blood circulating within the cranium is equal to 10—5 in the veins, and 5 in the arteries; if one of these be increased to 6, the other must be diminished to 4, so that the same amount, 10, is always preserved. It follows, that when fluids are effused, blood extravasated, or tumours grow, a corresponding amount of fluid must be pressed out, or of brain absorbed, from the physical impossibility of the cranium holding more matter. At the same time, it must be evident that an increased or diminished amount of pressure may be exerted *on* the brain, proportioned to the power of the heart's contraction, the effect of which will be, not to alter the amount of fluids within the cranium, but to cause, using the words of Abercrombie, 'a change of circulation' there.

"Dr. Kellie performed numerous experiments on cats and dogs, in order to elucidate this subject. Some of these animals were bled to death by opening the carotid or femoral arteries, others by opening the jugular veins. In some the carotids were first tied, to diminish the quantity of blood sent to the brain, and the jugulars were then opened, with the view of emptying the vessels of the brain to the greatest possible extent; while, in others, the jugulars were first secured, to prevent as much as possible the return of the blood from the brain, and one of the carotids was then opened. He inferred, from the whole inquiry, which was conducted with extreme care, 'That we cannot, in fact, lessen, to any considerable extent, the quantity of blood within the cranium by arteriotomy or venesection; and that when, by profuse hemorrhages destructive of life, we do succeed in draining the vessels within the cranium of any sensible portion of red blood, there is commonly found an equivalent to this spoliation in the increased circulation or effusion of serum, serving to maintain the plentitude of the cranium.'

"Dr. Kellie made other experiments upon the effects of position immediately after death from strangulation or hanging. He also removed a portion of the unyielding walls of the cranium in some animals, by means of a trephine, and then bled them to death; and the differences between the appearances of the brain in these cases, and in those where the cranium was entire, were very great. One of the most remarkable of these differences was its shrunken appearance, in those animals in which a portion of the skull was removed, and the air allowed to gravitate upon its inner surface. He says:—'The brain was sensibly depressed below the cranium, and a space left, which was found capable of containing a teaspoonful of water.'

"It results from these inquiries, that there must always be the same amount of fluids within the cranium so long as it is uninjured. In morbid conditions these fluids may be blood, serum, or pus; but in health, as blood is almost the only fluid present (the cerebro-spinal fluid being very trifling), its quantity can undergo only very slight alterations. There are many circumstances, however, which occasion local congestions in the brain, and consequently unequal pressure on its structure, in which case another portion of its substance must contain less blood, so that the amount of the whole, as to quantity, is always preserved. These circumstances are mental emotions, hemorrhages, effusions of serum, and morbid growths. Such congestions, or local hyperæmias, in themselves constitute morbid conditions; and nature has, to a great extent, provided against their occurrence under ordinary circumstances, by the tortuosity of the arteries and the cerebro-spinal fluid, described by Magendie.

"The views now detailed had been very extensively admitted into pathology, when Dr. Burrows, of St. Bartholomew's Hospital, endeavoured to controvert them, first in the Lumleian lectures of 1843, and subsequently in a work published in 1846, entitled, 'On Disorders of the Cerebral Circulation, and on the Connexion between Affections of the Brain and Diseases of the Heart.' Dr. Burrows, however, evidently formed the most confused notions of the doctrine we are advocating; for, instead of stating it as propounded by its authors, he *actually misrepresented it*, as Dr. Reid pointed out. Thus, he is always combating the idea that bloodletting, position, strangulation, &c., cannot affect the *blood in the brain;* whereas the real proposition is, that they cannot alter the *fluids within the cranium*. By thus confounding blood with fluid, and brain with cranium, he has only contrived to overthrow a theory of his own creation.

"Dr. Burrows has brought forward several observations and experiments, which he considers opposed to the theory now advocated. His facts are perfectly correct. I myself have repeated his experiments on rabbits, and can confirm his descriptions. It is the inferences he draws from them that are erroneous. For the paleness which results from hemorrhage, and the difference observable in the colour of the brain, when animals, immediately after death, are suspended by their ears or by their heels, is explicable by the diminished number of coloured blood particles in the one case, and by their gravitation downwards in the other. That the amount of fluid within the cranium was in no way affected, is proved by the plump appearance of the brains figured by Dr. Burrows, and the total absence of that shrunken appearance so well described by Dr. Kellie.

"Neither does our observation of what occurs in asphyxia or apnœa, oppose the doctrine in question, as Dr. Burrows imagines, but rather confirms it. On this point the following observations by Dr. John Reid are valuable. He says:—'If any circumstance could produce congestion of the vessels within the cranium, it would be that of death by hanging; for then the vessels going to and coming from the brain are, with the exception of the vertebral arteries, compressed and then obstructed. These two arteries, which are protected by the peculiarity of their course through the foramina of the transverse processes of the cervical vertebræ, must continue for a time to force their blood upon the brain, while a comparatively small quantity only can escape by the veins. Indeed, the greater quantity of blood carried to the encephalon by the vertebrals, returns by the internal jugulars, and not by the vertebral veins, which are supplied from the occipital veins of the spinal cord; and the anastomoses, between the cranial and vertebral sinuses, could carry off a small quantity of the blood only, transmitted along such large arteries as the vertebrals. And yet it is well known that there is no congestion of the vessels within the cranium after death by hanging, however gorged the external parts of the head may be by blood and serum.' This is admitted by Dr. Burrows, although he endeavours to get rid of so troublesome a fact by a gratuitous hypothesis, which will not bear a moment's examination, but for the refutation of which I must refer to the works of Dr. Reid.*

"On the whole, whether we adopt the expressions of local congestion of change of circulation within the cranium (Abercrombie), or of unequal pressure (Burrows), our explanation of the *pathological* phenomena may be made equally correct, because each term implies pretty much the same thing. But if we imagine that venesection will enable us to diminish the amount of blood in the cerebral vessels, the theory points out that this is impossible, and that the effects of bleeding are explained by the influence produced on the heart, the altered pressure on the brain, exercised by its diminished contractions, and the change of circulation within the cranium thereby occasioned.

"I have entered somewhat fully into this theory, because, independent of its vast importance in a practical point of view, it is one which originated in, and has always been maintained by, the Edinburgh School of Medicine. Singular to say, notwithstanding the obvious errors and fallacies in Dr. Burrows' work, no sooner did it appear than the whole medical press of England and Ireland adopted its conclusions, and even Dr. Watson, in the last edition of his excellent work, also abandoned the theory of Monro, Kelly, and Abercrombie. But so far is this theory concerning the circulation within the cranium from being shaken by the attack of Dr. Burrows, that it may be said now to stand on a firmer basis than ever, owing to that attack having drawn forth the convincing reasoning and unanswerable arguments of so sound an anatomist, physiologist, and pathologist, as the late Dr. John Reid.

"(2.) *All the functions of the nervous system may be increased, perverted, or destroyed, according to the degree of stimulus or disease operating on its various parts.* Thus, as a general rule, it may be said, that a slight stimulus produces increased or perverted action; whilst the same stimulus, long continued or much augmented, causes loss of function. All the various stimuli, whether mechanical, chemical, electrical, or psychical, produce the same effects, and in different degrees. Circumstances influencing the heart's action, stimulating drinks or food, act in a like manner. Thus, if we take the effects of alcoholic drink, for the purpose of illustration, we observe that, as regards combined movements, a slight amount causes increased vigour and activity in the muscular system. As the stimulus augments in intensity, we see irregular movements occasioned, staggering, and inability of directing the limbs. Lastly, when the stimulus is excessive, there is complete inability to move, and the power of doing so is temporarily annihilated. With regard to sensibility and sensation, we observe cephalalgia, tingling, and heat of skin, tinnitus aurium, confusion of vision, muscæ volitantes, double sight, and lastly, complete insensibility and coma. As regards intelligence, we observe at first rapid flow of ideas, then confusion of mind, delirium, and lastly, sopor and perfect unconsciousness. In the same manner, pressure, mechanical irritation, and the various organic diseases, produce augmented, perverted or diminished function, according to the intensity of the stimulus applied, or amount of structure destroyed.

"Thus it has been shown, that excess or diminution of stimulus, too much or too little blood, very violent or very weak cardiac contractions, and inflammation or extreme exhaustion, will, so far as the nervous functions are concerned, produce similar alterations of motion, sensation, and intelligence.' Excessive hemorrhage causes muscular weakness, convulsions, and loss of motor power, perversions of all the sensations, and lastly, unconsciousness from syncope. Hence the general strength

---

* "Monthly Journal," August, 1846. "Physiological, Anatomical, and Pathological Researches," No. XXV.

of the frame cannot be judged of by the nervous symptoms, although the treatment of these will be altogether different, according as the individual is robust or weak, has a full or small pulse, &c. These similar effects on the nervous centres from apparently such opposite exciting causes, can, it seems to me, only be explained by the peculiarity of the circulation previously noticed. A change of circulation within the cranium takes place, and whether arterial or venous congestion occurs, pressure on the organ is equally the result. The importance of paying attention to this point in the treatment must be obvious.

(3.) *The seat of the disease in the nervous system influences the nature of the phenomena or symptoms produced.* It is a matter of very great importance to ascertain how far certitude in diagnosis may be arrived at, and the seat of the disease ascertained. On this subject it may be affirmed that, although clinical observation combined with pathology have done much, more requires to be accomplished. As a general rule, it may be stated, that disease or injury of one side of the encephalon, above the decussation in the medulla oblongata, especially influences the opposite side of the body; whilst, if the spinal cord be affected below the decussation, the influence produced is not crossed, but direct. It is said that some very striking exceptions have occurred to this rule, but these at any rate are remarkably rare. Besides, it has always appeared to me probable that, inasmuch as extensive organic disease, if occurring slowly, may exist without producing symptoms, whilst it is certain most important symptoms may be occasioned without organic disease, even these few exceptional cases are really not opposed to the general law. Then, as a general rule, it may be said that diseases of the brain proper are more especially connected with perversion and alteration of the intelligence; whilst disease of the cranial portion of the spinal cord and base of the cranium, are more particularly evinced by alterations of sensation and motion. In the vertebral portion of the cord, the intensity of pain and of spasm, or want of conducting power, necessary to sensation and voluntary motion, indicates the amount to which the motor and sensitive columns are affected. Further than this we can scarcely generalize with prudence, although there are some cases, as we shall subsequently see, where careful observation has enabled us to arrive at more positive results,

The fatality of lesions affecting various parts of the nervous centres varies greatly. Thus the hemispheres may be extensively diseased, often without injury to life, or even permanent alteration of function. Convulsions and paralysis are the common results of disease of the ganglia, in the cranial portion of the cord. The same results from lesion of the pons varolii. But this, if it affect the medulla oblongata, where the eighth pair originates, or injury to this centre itself, is almost always immediately fatal.

(4.) *The rapidity or slowness with which the lesion occurs influences the phenomena or symptoms produced.* It may be said as a general rule, that a small lesion, for instance a small hemorrhagic extravasation, occurring suddenly, and with force, produces, even in the same situation, more violent effects than a very extensive organic disease which comes on slowly. Here, however, much will depend upon the seat of the lesion. Very extraordinary cases are on record, where large portions of the nervous centres have been much disorganized, without producing anything like such violent symptoms as have been occasioned at other times by a small extravasation in the same place. Here again the nature of the circulation within the cranium offers the only explanation, for the encephalon must undergo a certain amount of pressure, if no time be allowed for it to adapt itself to a foreign body; whereas any lesion coming on slowly enables the amount of blood in the vessels to be diminished according to circumstances, whereby pressure is avoided.

(5.) *The various lesions and injuries of the nervous system produce phenomena similar in kind.* The injuries which may be inflicted on the nervous system, as well as the morbid appearances discovered after death, are various. For instance, there may be an extravasation of blood, exudation of lymph, a softening, a cancerous tumour, or tubercular deposit, and yet they give rise to the same phenomena, and are modified only by the circumstances formerly mentioned, of degree, seat, suddenness, &c. Certain nervous phenomena also are of a paroxysmal character, whilst the lesions supposed to occasion them are stationary or slowly increasing. It follows, that the effects cannot be explained by the nature of the lesions, but to something which they all have in common; and this, it appears to me, may consist of,—1st, Pressure with or without organic change; 2nd, More or less destruction or disorganization of nervous texture. Further, when we consider that the same nervous symptoms arise from irregularities in the circulation from increased as well as diminished action, sometimes when no appreciable change is found, as well as when disorganization has occurred, the theory of local congestions in the nervous centres seems to me the most consistent with known facts. That such local congestions do frequently occur during life, without leaving traces detectable after death, is certain; whilst the occurrence of molecular changes, or other hypothetical conditions which have been supposed to exist, have never yet been shown to take place under any circumstances.

---

